# Supplementary material for: Large male proboscis monkeys have larger noses but smaller canines
Source: Commun Biol. 2020 Sep 21;3:522. doi: 10.1038/s42003-020-01245-0 (PMC7506553; doi:10.1038/s42003-020-01245-0)
Supplement: Supplementary file 1 — Supplementary Information [file 42003_2020_1245_MOESM1_ESM.pdf]

Supplementary Information

Large male proboscis monkeys have larger noses but smaller canines

Ikki Matsuda[1,2,3,4*], Danica J. Stark[5], Diana A. Ramirez Saldivar[5,6], Augustine Tuuga[6], Senthilvel K. S. S. Nathan[6], Benoit Goossens[5,6,7,8], Carel P. van Schaik[9], Hiroki Koda[10*]

[1]Chubu University Academy of Emerging Sciences, 1200, Matsumoto-cho, Kasugai-shi, Aichi 487-8501, Japan
[2]Wildlife Research Center of Kyoto University, Kyoto, Japan
[3]Japan Monkey Centre, Inuyama, Japan
[4]Institute for Tropical Biology & Conservation, Universiti Malaysia, Sabah, Malaysia
[5]Danau Girang Field Centre, c/o Sabah Wildlife Department, Sabah, Malaysia
[6]Sabah Wildlife Department, Sabah, Malaysia
[7]Sustainable Places Research Institute, Cardiff University, Cardiff, UK
[8] Organisms and Environment Division, Cardiff School of Biosciences, Cardiff University, Cardiff, UK
[9]Anthropological Institute and Museum, University of Zurich, Zurich, Switzerland
[10]Primate Research Institute, Kyoto University, Inuyama, Aichi 484-8506, Japan

*Correspondence: ikki-matsuda@isc.chubu.ac.jp (IM) and koda.hiroki.7a@kyoto-u.ac.jp (HK)

Supplementary Table 1. List of subject information. All study subjects were adults, i.e., 18 harem-holding males and 10 females including one pregnant and two lactating individuals

Sex	Age/class	Nose size (cm, square root)	Body mass (kg, cube root)	Maxillary canine length (mm)	Note
Male 1	Adult	5.7	2.7	17.0	
Male 2	Adult	5.7	2.8	20.5	
Male 3	Adult	5.7	2.8	18.6	
Male 4	Young adult	6.2	2.8	20.0	
Male 5	Old adult	6.1	2.9	17.0	
Male 6	Adult	5.9	2.7	22.1	
Male 7	Old adult	5.8	2.8	17.2	
Male 8	Adult	6.3	2.9	15.1	
Male 9	Adult	6.0	2.7	20.0	
Male 10	Adult	5.9	2.9	13.2	
Male 11	Adult	5.3	2.8	14.2	
Male 12	Adult	5.9	2.8	22.4	
Male 13	Adult	6.0	2.8	20.3	
Male 14	Adult	6.3	2.9	18.3	
Male 15	Adult	6.2	2.8	12.2	
Male 16	Adult	6.1	2.7	19.8	
Male 17	Adult	6.1	2.7	24.2	
Male 18	Young adult	5.6	2.7	20.0	
Female 1	Adult	3.5	2.4	5.5	
Female 2	Adult	3.2	2.3	7.6	Pregnant
Female 3	Adult	3.5	2.4	8.2	
Female 4	Adult	3.6	2.4	8.0	Lactating
Female 5	Adult	3.5	2.4	9.3	
Female 6	Adult	3.3	2.3	7.2	
Female 7	Young adult	3.2	2.2	9.6	
Female 8	Adult	3.1	2.2	8.3	
Female 9	Adult	2.8	2.1	7.0	Lactating
Female 10	Adult	3.7	2.2	8.6	
