# Supplementary material for: Large male proboscis monkeys have larger noses but smaller canines
Source: Commun Biol. 2020 Sep 21;3:522. doi: 10.1038/s42003-020-01245-0 (PMC7506553; doi:10.1038/s42003-020-01245-0)
Supplement: Supplementary file 3 — Reporting Summary [file 42003_2020_1245_MOESM3_ESM.pdf]

nature research

Corresponding author(s): Ikki Matsuda & Hiroki Koda

Last updated by author(s): Jun 10, 2020

Reporting Summary

Nature Research wishes to improve the reproducibility of the work that we publish. This form provides structure for consistency and transparency in reporting. For further information on Nature Research policies, see Authors & Referees and the Editorial Policy Checklist .

Statistics

For all statistical analyses, confirm that the following items are present in the figure legend, table legend, main text, or Methods section.

n/a	Confirmed	
☐	☒	The exact sample size (*n*) for each experimental group/condition, given as a discrete number and unit of measurement
☐	☒	A statement on whether measurements were taken from distinct samples or whether the same sample was measured repeatedly
☐	☒	The statistical test(s) used AND whether they are one- or two-sided<br>*Only common tests should be described solely by name; describe more complex techniques in the Methods section.*
☐	☒	A description of all covariates tested
☐	☒	A description of any assumptions or corrections, such as tests of normality and adjustment for multiple comparisons
☐	☒	A full description of the statistical parameters including central tendency (e.g. means) or other basic estimates (e.g. regression coefficient) AND variation (e.g. standard deviation) or associated estimates of uncertainty (e.g. confidence intervals)
☐	☒	For null hypothesis testing, the test statistic (e.g. *F*, *t*, *r*) with confidence intervals, effect sizes, degrees of freedom and *P* value noted<br>*Give P values as exact values whenever suitable.*
☒	☐	For Bayesian analysis, information on the choice of priors and Markov chain Monte Carlo settings
☒	☐	For hierarchical and complex designs, identification of the appropriate level for tests and full reporting of outcomes
☐	☒	Estimates of effect sizes (e.g. Cohen's *d*, Pearson's *r*), indicating how they were calculated

Our web collection on statistics for biologists contains articles on many of the points above.

Software and code

Policy information about availability of computer code

Data collection	No software was used in data collection.
Data analysis	R (version 3.3.2); python3 (matplotlib3.0.1, numpy 1.15.2, pandas 0.23.4, python 3.6.7, seaborn 0.9.0)

For manuscripts utilizing custom algorithms or software that are central to the research but not yet described in published literature, software must be made available to editors/reviewers. We strongly encourage code deposition in a community repository (e.g. GitHub). See the Nature Research guidelines for submitting code & software for further information.

Data

Policy information about availability of data

All manuscripts must include a data availability statement. This statement should provide the following information, where applicable:

- Accession codes, unique identifiers, or web links for publicly available datasets
- A list of figures that have associated raw data
- A description of any restrictions on data availability

Mathematical models and parameter proposals are described in the main text or code repositories (https://github.com/hkoda/nasalis_canine_bodymass_model), and data in support of the findings of this study are available from the corresponding authors by reasonable request.

Field-specific reporting

Please select the one below that is the best fit for your research. If you are not sure, read the appropriate sections before making your selection.

☐ Life sciences ☐ Behavioural & social sciences ☒ Ecological, evolutionary & environmental sciences

Ecological, evolutionary & environmental sciences study design

All studies must disclose on these points even when the disclosure is negative.

Study description	We investigated the mechanism underlying the trade-off among body mass, nose size, and canine size in free-ranging proboscis monkeys in Sabah, Borneo, Malaysia. Whereas male nose size had a positive correlation with body size, we unexpectedly found a negative correlation between body and canine sizes. Similar relationship between nose and body size was observed in females, whereas only a weak correlation was noted between canine and body size. We explain this by an interaction between sexual and natural selection. Larger noses in males may interfere with the use of canines, thereby reducing their effectiveness as weapons. However, longer canines are opposed by natural selection because they impose a larger gape upon its bearer and reduce foraging efficiency, particularly in folivores. The difference between the sexes regarding the regression intercept for body size vs. canine size was significant, although the difference of the slopes was insignificant, consistent with the hypothesis that the nose interferes with the effectiveness of canines as weapons in males but not in females. Moreover, a mathematical simulation supported our prediction. This unique case of decoupling of body and canine size reveals that large canines carry an ecological cost.
Research sample	18 and 10 free-ranging adult male and female proboscis monkeys, respectively.
Sampling strategy	No predetermined sample size.
Data collection	We captured free-ranging adult male proboscis monkeys, as described in detail in the methods and ethical statement of Stark et al. (2017). In this study, we added the data for maxillary canine length to those on body mass and nose size (length × width) for the same males obtained by Koda et al. (2018) as well as data for female canine length, body mass, and nose size.
Timing and spatial scale	Capturing and measuring animals occurred from August 2015 - 2016 in the Lower Kinabatangan Floodplain, Sabah, Borneo, Malaysia (5°18′N to 5°42′N and 117°54′E to 18°33′E).
Data exclusions	No data excluded from the analysis.
Reproducibility	N/A
Randomization	N/A
Blinding	N/A

Did the study involve field work? ☒ Yes ☐ No

Field work, collection and transport

Field conditions	Tropical forest habitat along river
Location	Lower Kinabatangan Floodplain, Sabah, Borneo, Malaysia (5°18′N to 5°42′N and 117°54′E to 18°33′E)
Access and import/export	All fieldwork was conducted in accordance with Malaysian laws and regulations. Permission was provided by the Sabah Biodiversity Centre, permit JKM/MBS.1000-2/2 JLD 3 (73), granted on 12/03/2015, arranged through the Sabah Wildlife Department.
Disturbance	All animal handling was carried out in accordance with the current laws of Malaysia and the Sabah Wildlife Department's Standard Operation Procedures on Animal Capture, Anaesthesia and Welfare.

Reporting for specific materials, systems and methods

We require information from authors about some types of materials, experimental systems and methods used in many studies. Here, indicate whether each material, system or method listed is relevant to your study. If you are not sure if a list item applies to your research, read the appropriate section before selecting a response.

Materials & experimental systems

n/a	Involved in the study	
☒	☐	Antibodies
☒	☐	Eukaryotic cell lines
☒	☐	Palaeontology
☐	☒	Animals and other organisms
☒	☐	Human research participants
☒	☐	Clinical data

Methods

n/a	Involved in the study	
☒	☐	ChIP-seq
☒	☐	Flow cytometry
☒	☐	MRI-based neuroimaging

Animals and other organisms

Policy information about studies involving animals; ARRIVE guidelines recommended for reporting animal research

Laboratory animals	N/A
Wild animals	We captured free-ranging adult male proboscis monkeys. All animal handling was carried out in accordance with the current laws of Malaysia and the Sabah Wildlife Department's Standard Operation Procedures on Animal Capture, Anaesthesia and Welfare [see in the main text for detailed statements]. The animals were anesthetized with Zoletil 100 (tiletamine + zolazepam, 6 ± 10 mg/kg), and a prophylactic dose of alamycin LA (20 mg/kg) and ivermectin (0.2 mg/kg) was given as a preventive measure to assist in postanesthesia recovery. Anesthesia and vital signs were monitored throughout the procedure. Forestomach contents were collected using catheter stomach tubes (Fr. 18 or 6.0 mm) for humans (TERMO®: JMDN code 70232000) while the animal was anesthetized.
Field-collected samples	N/A
Ethics oversight	No ethical oversight required.

Note that full information on the approval of the study protocol must also be provided in the manuscript.
